# Multidimensional regulation of hepatic macrophages in chronic liver disease: from metabolic reprogramming to intercellular and interorgan crosstalk

**DOI:** 10.3389/fimmu.2026.1953712

**Published:** 2026-09-29

**Authors:** Che Xu, Xin-Yu Zhu, Hai-Na Xie, Jing An, Jun-Feng Zhu, Si-Cheng Gao

**Affiliations:** 1 The Department of Hepatology, Yueyang Hospital, Shanghai University of Traditional Chinese Medicine, Shanghai, China; 2 The Department of Nephrology, Yueyang Hospital, Shanghai University of Traditional Chinese Medicine, Shanghai, China

**Keywords:** chronic liver diseases, exosomes, hepatic macrophages, heterogeneity, metabolic reprogramming, neuro-immune regulation

## Abstract

Chronic liver diseases (CLDs), including metabolic dysfunction-associated steatotic liver disease (MASLD), alcohol-associated liver disease (ALD, viral hepatitis, liver fibrosis, cirrhosis and hepatocellular carcinoma (HCC)), impose enormous global public health burdens. Hepatic macrophages act as core orchestrators governing the initiation and progression of all CLD subtypes. This review systematically integrates recent advances regarding hepatic macrophage ontogeny, spatial heterogeneity, metabolic rewiring, multi-cellular communication, and systemic neuro-immune crosstalk along the gut-liver-brain axis. Beyond the outdated binary M1/M2 classification, tissue-resident macrophages (ResMø) and recruited monocyte-derived macrophages (MDMs) differentiate into disease-specific subsets (LAMs, SAMs, SenAMs) with spatially restricted, context-dependent functions. Metabolic reprogramming-glycolysis for pro-inflammatory polarization versus oxidative phosphorylation (OXPHOS)/fatty acid oxidation (FAO) for tissue-repair phenotypes-serves as the intrinsic molecular switch shaping macrophage effector profiles. Extrinsically, bidirectional signaling between hepatic macrophages and hepatocytes, hepatic stellate cells (HSCs), liver sinusoidal endothelial cells (LSECs), and other intrahepatic immune cells dictates the balance between hepatic injury and tissue regeneration. Macrophage-derived autophagy, LC3-associated phagocytosis (LAP), and exosomal cargo transfer represent pivotal functional executors mediating intercellular signal transmission. Furthermore, gut microbial metabolites and bidirectional liver-brain neuroimmune signals converge on hepatic macrophages, linking local hepatic inflammation to systemic metabolic and neurocognitive complications. This review further summarizes unresolved bottlenecks and translational prospects for macrophage-targeted precision therapy. Collectively, these multi-layered regulatory networks provide a comprehensive theoretical framework for identifying novel therapeutic targets across the full spectrum of CLDs.

## 1 Introduction

Chronic liver disease (CLD) is defined as persistent progressive hepatic dysfunction lasting over six months, manifested by impaired synthetic capacity, defective detoxification, and disrupted bile excretion. CLD inevitably advances into irreversible fibrosis, decompensated cirrhosis and HCC, with MASLD, ALD and chronic viral hepatitis constituting the three dominant etiological categories (1, 2). Global burden data demonstrated that cirrhosis and related liver pathologies caused 1.26 million deaths worldwide in 2019, with a 13% mortality increase compared to 1990 (3). Current therapeutic regimens remain incomplete: while antiviral agents effectively suppress HBV/HCV replication, Resmetirom and Saroglitazar have only recently obtained approval for MASLD, and no FDA-specific therapeutics are available for ALD or advanced liver fibrosis (4, 5). There exists an urgent unmet clinical demand to uncover core pathogenic drivers for developing targeted interventions.

Macrophages, abundant innate immune cells, play pivotal roles in tissue homeostasis and disease (6). Hepatic macrophages mainly comprise two developmentally distinct but functionally complementary populations. One population consists of tissue-resident macrophages, classically referred to as Kupffer cells, which are predominantly derived from embryonic yolk sac progenitors and possess self-renewal capacity within the liver. The other comprises monocyte-derived macrophages (MDMs), which are recruited from circulating monocytes and differentiate within the liver in response to pathological conditions, including hepatic injury, inflammation, and metabolic stress. These two macrophage populations exhibit context-dependent and complementary functions in maintaining hepatic homeostasis, coordinating responses to tissue injury, and promoting tissue repair. Through their dynamic interactions, tissue-resident macrophages(ResMø) and MDMs contribute to hepatic immune surveillance and regulation of the local microenvironment, thereby constituting an essential immunological barrier for both tissue protection and homeostatic maintenance (7).The conventional M1/M2 polarization dichotomy has proven oversimplified to recapitulate the full spectrum of macrophage functional plasticity, as cellular origin, anatomical zonation, and local microenvironmental cues jointly reshape their transcriptomic and functional landscapes (8, 9). Single-cell and spatial transcriptomic approaches have further uncovered stage-specific subsets in CLD, including lipid-associated macrophages (LAMs), scar-associated macrophages (SAMs), and senescent macrophages (SenAMs), each exerting stage-specific pathogenic or protective roles (10).

Herein, we construct a three-axis integrated paradigm to dissect hepatic macrophage regulation in CLD: (1) intrinsic metabolic reprogramming controlling macrophage polarization; (2) intrahepatic intercellular crosstalk coordinating injury and repair; (3) systemic gut-liver-brain neuroimmune communication linking hepatic lesions to whole-body complications. We further highlight autophagy, LAP and exosomal signaling as core functional mediators bridging these three regulatory layers and conclude with outstanding scientific challenges and translational therapeutic strategies targeting macrophage ecosystem remodeling. This review is supported by the research program focused on heat-clearing, dampness-disinhibiting, turbidity-resolving and lipid-purifying standardized therapy for damp-heat syndrome MAFLD (24Y12801100), providing immunological evidence for traditional Chinese medicine (TCM) intervention against metabolic liver injury.

## 2 Heterogeneity and dynamic evolution of hepatic macrophages

### 2.1 Macrophage subsets under physiological homeostasis

In steady-state healthy liver, ResMø anchored within hepatic sinusoids represent the dominant macrophage population, marked by specific surface markers CLEC4F, VSIG4 and CLEC2 in murine models (11–13). ResMø subpopulations exhibit intrinsic functional segregation: CD68^+^ ResMø subsets specialize in phagocytosis of circulating debris, while CD11b^+^ counterparts prioritize cytokine secretion to sustain hepatic immune tolerance via constitutive IL-10 and TGF-β release (14). Additional minor resident macrophage populations include liver capsular macrophages (LCMs) residing in outer hepatic connective tissues and periportal CX3CR1^+^CD63^+^ portal-associated macrophages (LPAMs) positioned adjacent to hepatic sympathetic nerve plexuses (15–17). LPAMs undertake antigen presentation and maintain portal immune surveillance, whereas pericentral CX3CR1^+^ macrophages regulate sinus vascular remodeling under physiological conditions (16).

Circulating monocytes barely infiltrate intact hepatic parenchyma under homeostasis, and MDM accumulation only occurs upon tissue damage. Spatial zonation strictly compartmentalizes macrophage functions across liver lobules, forming a spatially layered immune surveillance network to intercept gut-derived microbial products delivered via the portal vein (**Figure 1**).

**Figure 1:**
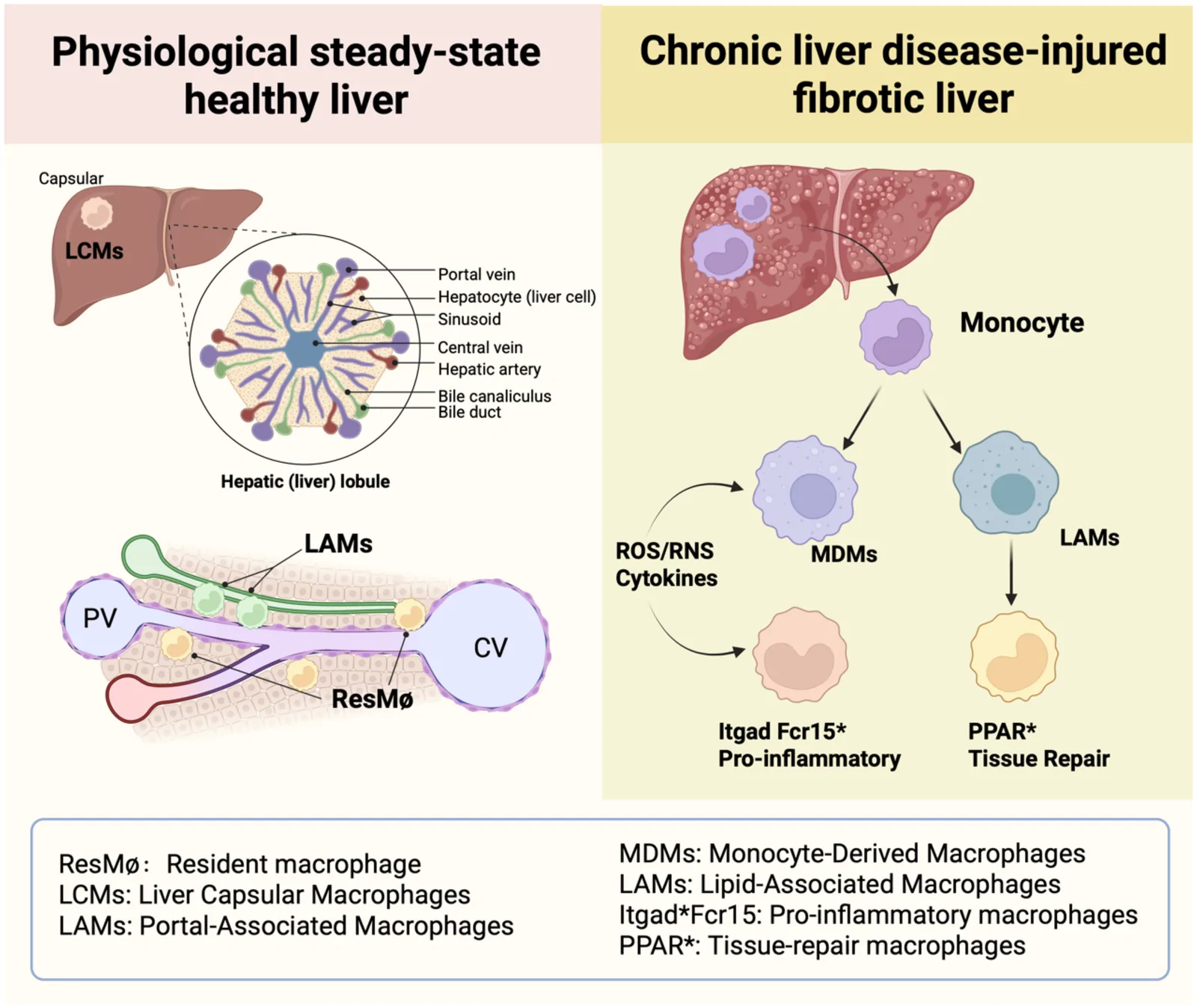
Heterogeneity and dynamic evolution of hepatic macrophages in health and chronic liver disease. Schematic overview of hepatic macrophage ontogeny, spatial distribution, and disease-associated subset remodeling. In the steady-state healthy liver (left), the magnified inset depicts a classic hepatic lobule with well-defined portal triad, central vein, sinusoids, and hepatocyte cords; sinusoid-resident macrophages are labeled as Kupffer cells (KCs), together with LCMs and LAMs. Upon chronic injury (right), recruited monocyte-derived macrophages (MDMs) give rise to pathological subsets—including LAMs, SAMs, SenAMs, and TAMs—in a disease-stage-dependent manner, highlighting the transition from the binary M1/M2 model to a dynamic macrophage landscape.

### 2.2 Remodeling of macrophage landscape during CLD progression

Upon chronic hepatic insult, ResMø undergo massive depletion, with circulating monocytes massively recruited to differentiate into pathogenic MDM subsets that redefine intrahepatic immune architecture. Disease etiology dictates the spatial localization and transcriptomic signature of MDM-derived specialized macrophages: in biliary tract disorders, Trem2^+^Spp1^+^ SAMs aggregate around portal triads to drive periportal fibrosis; in MASLD/MASH, LAMs accumulate within pericentral lobular regions to amplify lipotoxic inflammation and fibrogenesis. Single-cell transcriptomics identified Itgad^+^Fcrl5^+^ pro-inflammatory macrophage subclusters that propagate inflammatory cascades via chemokine and pro-cytokine release, while Trem2^+^ApoE^+^ LAMs exert dual effects: early-stage hepatoprotection and late-stage profibrotic transformation (18, 19).

Senescence-associated macrophages (SenAMs, p16INK4a^+^) constitute another critical pathogenic subset across fibrosis and HCC. SenAMs secrete TGF-β and PDGF to activate HSCs and accelerate collagen deposition; in the HCC microenvironment, senescent endothelial cells recruit mesenchymal progenitors to drive cholangiocytic transdifferentiation of tumor cells, reshaping the tumor immune microenvironment (20, 21). Tumor-associated macrophages (TAMs, particularly CCL18^+^ subsets) facilitate tumor immune escape by suppressing cytotoxic CD8^+^ T cell activity, while PDGFRA^+^ cancer-associated fibroblasts further polarize TAMs toward pro-neoplastic phenotypes (22).

Functionally, activated hepatic macrophage release pro-inflammatory mediators (TNF-α, IL-1β, IL-12), reactive oxygen species(ROS)and reactive nitrogen species(RNS) to amplify hepatocyte injury, yet simultaneously mediate apoptotic cell clearance and systemic iron, cholesterol and bilirubin homeostasis (23–26). Pro-inflammatory macrophage sequester intracellular iron to limit local immune suppression, while ResMø dominate hepatic lipid uptake and oxidation to maintain metabolic equilibrium independent of MDM-mediated inflammatory responses (27). Collectively, hepatic macrophage identity has transitioned from a static binary classification to a spatially organized, disease-stage-dependent plastic cellular network.

## 3 Metabolic reprogramming as the intrinsic switch of macrophage polarization

### 3.1 Distinct metabolic states sustain distinct macrophage functional phenotypes

Macrophage polarization is irreversibly coupled to wholesale rewiring of central carbon, lipid and mitochondrial metabolism (**Figure 2**). Upon stimulation with lipopolysaccharide(LPS)/Toll-like receptor 4 and other inflammatory signals, pro-inflammatory macrophages undergo a profound metabolic reprogramming characterized by markedly enhanced glycolytic flux and concomitant activation of *de novo* fatty acid synthesis. This metabolic configuration endows inflammatory macrophages with the capacity for rapid ATP generation, which serves as the reducing equivalent for the synthesis of reactive oxygen species and nitric oxide, thereby augmenting their microbicidal activity. Meanwhile, upregulated pyruvate dehydrogenase kinase (PDK) blocks pyruvate mitochondrial import, diverting carbon flux to lactate production; accumulated lactate and succinate stabilize hypoxia-inducible factor 1α(HIF-1α)and activate nuclear factor kappa-B(NF-κB)signaling to drive transcription of pro-inflammatory cytokines (IL-6, TNF-α). Additionally, through the secretion of chemokines, these macrophages recruit neutrophils, monocytes, and other immune cells to sites of infection or tissue injury, thereby amplifying and orchestrating the inflammatory cascade, and playing a pivotal role in host anti-infective immune responses (28–30). Notably, with the advent of high-resolution macrophage capture and sorting technologies, researchers have increasingly recognized that macrophage subsets with glycolysis as their central metabolic hallmark can sense microenvironmental cues and orchestrate inflammatory signaling crosstalk, thereby exerting pro-pathological roles in the progression of chronic liver diseases. Yang CQ et al. demonstrated that FSP1^+^macrophage subsets regulate pyruvate kinase M2(PKM2)stability in a glycolysis-dependent manner, thereby driving lactylation of PGK1 and establishing a self-reinforcing positive feedback loop that accelerates hepatic fibrosis (31). Ling Q et al. revealed that hepatocyte-derived sphingosine-1-phosphate promotes PKM2 dimerization and subsequent nuclear translocation, which in turn coordinates pro-inflammatory phenotypic polarization of macrophages through the PKM2/HIF-1α axis, ultimately contributing to the pathogenesis of MASH (32).

**Figure 2:**
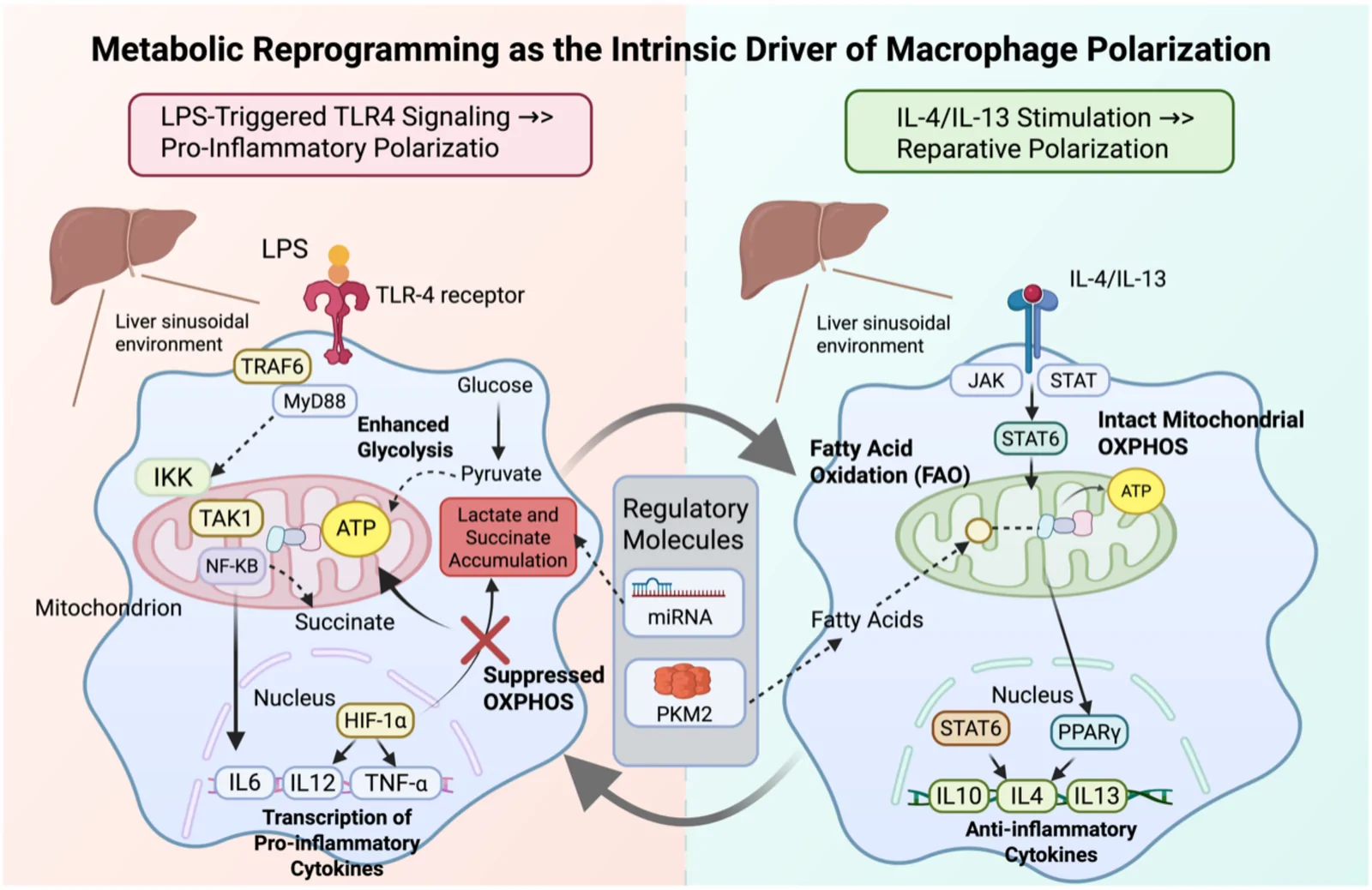
Metabolic reprogramming as the intrinsic driver of macrophage polarization. Divergent metabolic pathways underlying pro-inflammatory and tissue-repair macrophage phenotypes. Pro-inflammatory macrophages exhibit enhanced glycolysis with HIF-1α, PKM2, and NF-κB activation, leading to lactate production and pro-inflammatory cytokine secretion. Tissue-repair macrophages rely on OXPHOS and FAO, regulated by PPARγ, to support IL-10 secretion and tissue remodeling. Key metabolic checkpoints are highlighted as potential therapeutic nodes.

Multiple metabolic-sensing pathways—including HIF-1α, succinate, lactate, PDK, PKM2, and TLR4-NF-κB—coordinate macrophage polarization, but their causal roles in liver disease vary substantially by cell type, disease stage, and experimental model (summarized in **Table 1**).

**Table 1:** Evidence matrix for macrophage-centered metabolic and signaling pathways in chronic liver disease.

Pathway or mediator	Mediated metabolic reprogramming in macrophages	Principal macrophage-centered role	Evidence tier
HIF-1α (33, 34)	Drives aerobic glycolysis; suppresses mitochondrial OXPHOS; promotes hypoxic adaptive metabolism	Hypoxic adaptation and glycolytic inflammatory activation.	Tier I in macrophage immunometabolism; Tier II in chronic liver disease
Succinate (35–37)	Stabilizes HIF-1α; promotes mitochondrial ROS production; mediates extracellular signaling via SUCNR1	Intracellular HIF-1α/ROS signaling and extracellular SUCNR1 signaling.	Tier II (cell- and model-dependent)
Lactate and protein lactylation (38)	Lactate transport via MCT; activates HCAR1 signaling; mediates histone and non-histone lactylation	MCT/HCAR1 signaling and histone or nonhistone lactylation.	Tier II mechanistically; Tier III for liver-specific causality
PDK family (particularly PDK1) (34)	Phosphorylates PDH to inhibit pyruvate entry into the TCA cycle; enforces glycolytic adaptation to hypoxia	Pyruvate dehydrogenase inhibition and hypoxic glycolytic adaptation.	Tier I for hypoxic migration; Tier II in chronic liver disease
PKM2 (39, 40)	Enters nucleus as a dimer to activate HIF-1α transcription; regulates glycolytic flux; regulated by oligomeric switching	Glycolytic flux and HIF-1α-dependent transcription through oligomeric switching.	Tier I in activated macrophages; Tier II in chronic liver disease
TLR4-NF-κB (41, 42)	Senses PAMPs/DAMPs; activates NF-κB inflammatory transcriptional network	PAMP/DAMP sensing and inflammatory cytokine signaling.	Tier I, with cell- and phase-specific effects
JAK-STAT signaling (43, 44)	Mediates cytokine-driven transcriptional reprogramming; STAT1 pro-inflammatory, STAT3 predominantly anti-inflammatory/repair	Cytokine-driven inflammatory, antimicrobial, and restorative states.	Tier I for defined modules; Tier II as an aggregate pathway
TGF-β-SMAD2/3 (45)	Macrophages secrete TGF-β ligand; signals via SMAD2/3 to drive pro-fibrotic transcription	HSC activation and matrix production; macrophage-derived ligand supply.	Tier I for HSC fibrogenesis; Tier II for macrophage-HSC crosstalk
Extracellular vesicles and specific miRNAs	Mediates intercellular transfer of metabolites, signaling proteins, and nucleic acids (miRNAs, dsRNA) via EVs	Intercellular transfer of regulatory cargo.	Tier III (emerging)

Evidence tiers: Tier I, direct genetic and/or convergent *in vivo* evidence; Tier II, mechanistic evidence limited by cell specificity, model, or pharmacologic inference; Tier III, predominantly associative, *in vitro*, overexpression, or delivery-based evidence. Grades apply only to the specified cell type, model, and disease phase.

Conversely, stimulation with interleukin-4 (IL-4) and interleukin-13 (IL-13) polarizes macrophages toward an anti-inflammatory phenotype endowed with robust tissue reparative functions. These macrophages are critically dependent on functionally intact mitochondrial oxidative phosphorylation (OXPHOS) and sustained fatty acid oxidation (FAO). This metabolic reprogramming is precisely coordinated by the nuclear receptor peroxisome proliferator-activated receptorγ (PPARγ), which integrates lipid metabolic signals to drive fatty acid transport into mitochondria and their subsequent oxidative catabolism (46, 47). In this progress, PKM2 restrains pro-inflammatory transcriptional programs to promote anti-inflammatory IL-10 secretion and extracellular matrix formation during injury resolution. However, persistent inflammatory and tissue injury stimuli can induce metabolic dysregulation in these macrophages. Excessively enhanced fatty acid oxidation generates ATP to meet the intrinsic energy demands of Mø; concurrently, the associated paracrine effects promote the activation and myofibroblastic transdifferentiation of HSCs. Notably, this process is accompanied by mitochondrial damage and a secondary metabolic switch toward glycolysis in macrophages, which synergistically establish a self-amplifying positive feedback loop that perpetually drives fibrosis progression (48–50). Currently, macrophages subsets characterized by OXPHOS as their core metabolic paradigm have transcended the binary classification framework of conventional M2 macrophages, achieving increasingly refined molecular delineation. Accumulating evidence indicates that this metabolic phenotype can be further stratified into functionally distinct subpopulations. For instance, SPP1^+^macrophage subsets exert pronounced pro-fibrotic effects through sustained OXPHOS coupled with FAO, whereas triggering receptor expressed on TREM2^+^ macrophage subsets facilitate tumor immune evasion via mitochondrial oxidative metabolism (51, 52). Moving forward, future investigations should integrate single-cell multi-omics technologies with metabolic tracing approaches to systematically dissect the metabolic–functional coupling mechanisms of distinct OXPHOS-dependent macrophage subsets within specific tissue microenvironments. Concurrently, the development of precision therapeutic strategies targeting specific subsets based on their metabolic heterogeneity holds considerable promise for advancing the treatment of chronic liver diseases, malignancies, and fibrotic disorders.

### 3.2 Mitochondrial dysfunction: core driver of lipotoxicity and inflammation

Lipid metabolic dysregulation represents the central pathogenic node linking macrophage metabolic reprogramming to MASLD progression (**Figure 3**). Excessive free fatty acid uptake triggers endoplasmic reticulum (ER) stress and shifts resident macrophage phenotypes toward pro-inflammatory M1 profiles (53). THRSP^+^ lipotoxic hepatocytes recruit CD74^+^ LAMs via MIF signaling in periportal zones, while lipid droplet cholesterol accumulation establishes a vicious lipotoxic cycle characterized by mitochondrial damage, mitochondrial DNA (mtDNA) leakage and elevated ROS production (54). Two functionally distinct mitochondrial pools coordinate lipid handling: cytoplasmic mitochondria (CMs) mediate FAO, whereas lipid droplet-associated mitochondria (LDMs) facilitate lipid esterification (55); LDM functional impairment in MASLD exacerbates lipotoxic damage and inflammasome activation (56). Triggering receptor expressed on myeloid cells 2/membrane-spanning 4-domains subfamily A member 7 (TREM2/MS4A7) -dependent lipid droplet efferocytosis mitigates this inflammatory cascade by clearing lipid-laden apoptotic cellular debris (57).

**Figure 3:**
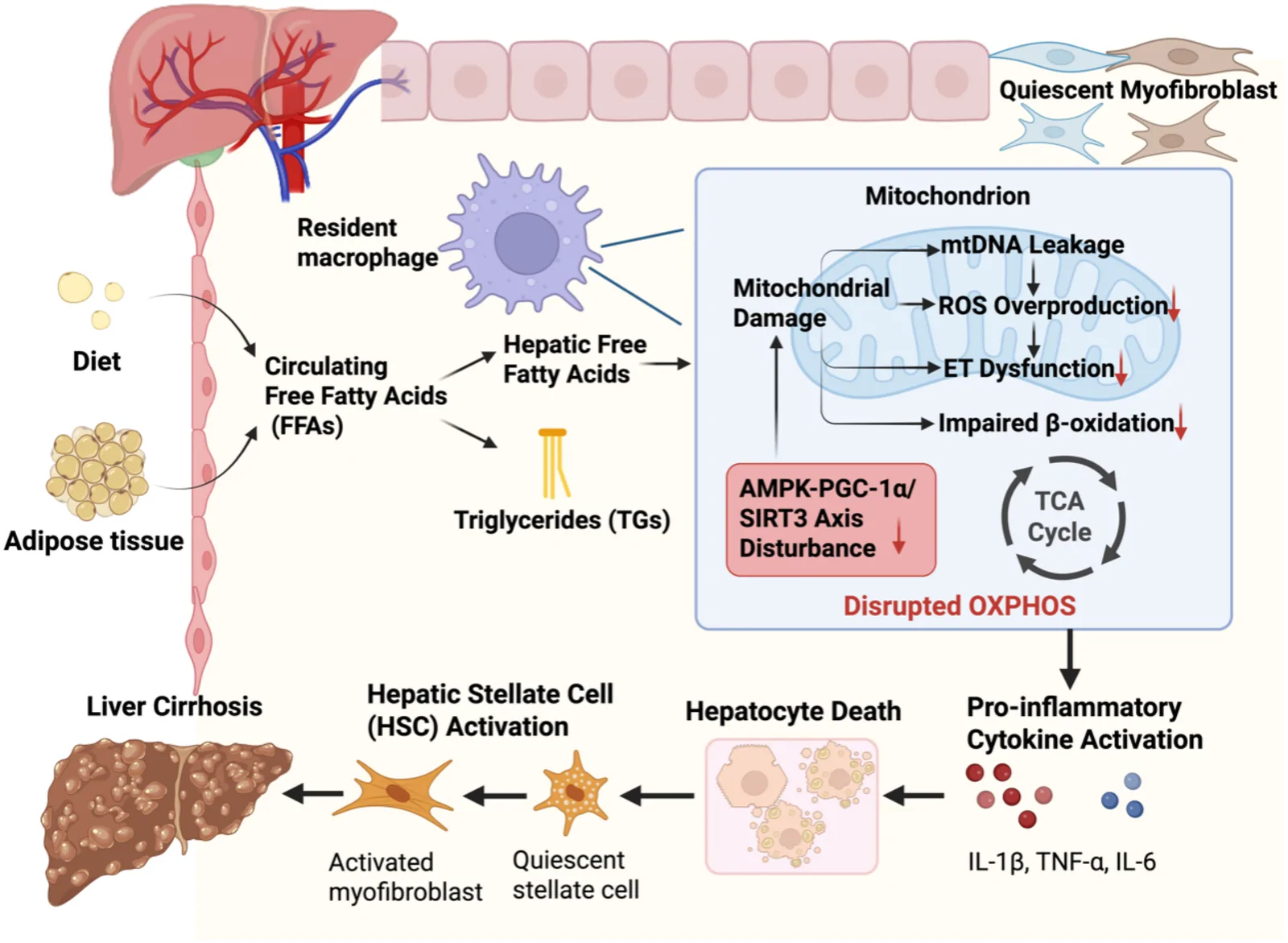
Mitochondrial dysfunction and lipid metabolic crosstalk in macrophage-driven liver injury. Mitochondrial dysfunction links lipid overload to macrophage-mediated inflammation. Excessive free fatty acids trigger ER stress, mitochondrial damage, mtDNA release, and ROS production, activating NLRP3 inflammasomes and pro-inflammatory polarization. CMs and LDMs coordinate lipid handling; their impairment exacerbates lipotoxicity. TREM2/MS4A7-dependent efferocytosis serves as a counter-regulatory mechanism.

Mitochondrial structural and functional breakdown further propagates CLD across all etiologies. Hypoxia-induced HIF-1α signaling enforces glycolytic M1 polarization, while acetaldehyde (ALD metabolite) inhibits mitochondrial complex I to disrupt nicotinamide adenine dinucleotide (NAD^+^/NADH) balance and boost pro-inflammatory cytokine secretion (58, 59). In HCC, TAMs utilize aerobic glycolysis to produce lactate, suppressing intertumoral anti-tumor T cell immunity; Golgi membrane protein 1- ATP citrate lyase (GOLM1-ACLY) signaling further amplifies macrophage metabolic reprogramming to accelerate tumor proliferation (60).Amino acid metabolic competition adds another regulatory layer: M2 macrophages utilize arginase to degrade collagen during fibrosis regression, whereas HCC tumor cells deplete microenvironmental glutamine to skew macrophage toward pro-neoplastic profiles via mTOR activation. Mitochondrial fission-fusion imbalance releases mtDNA to activate NLRP3 inflammasomes, while methylation-controlled J protein (MCJ) mitochondrial protein inhibition alleviates oxidative injury in ALD and cholestatic liver disease (61–63).

## 4 Multi-layered intercellular and systemic crosstalk mediated by hepatic macrophages

### 4.1 Intrahepatic cell-cell communication network

Hepatic macrophages act as central signal hubs coordinating bidirectional communication with parenchymal and non-parenchymal hepatic cell populations (**Figure 4**). Injured hepatocytes release high mobility group box 1 (HMGB1) and other damage-associated molecular patterns (DAMPs) to activate macrophage TLR4/NF-κB inflammatory signaling; ferroptosis suppression reduces DAMP release and attenuates subsequent hepatic inflammation (64, 65). Macrophages exert dual regulatory effects on hepatocyte fate: metabolically rewired MDMs utilize glutamate-HIF1α-WNT3-YAP1 signaling to promote hepatocyte proliferation and liver regeneration, while pathological M1 macrophages secrete TNF-α and IL-6 to exacerbate insulin resistance and lipotoxic steatosis (66). ResMø and hepatocytes cooperatively induce inhibitor of DNA binding 3 (ID3) expression to restrain overactive macrophage inflammation, while LSECs and HSCs establish ResMø niche identity through NOTCH-BMP-dependent LXR-α upregulation (67–69).

**Figure 4:**
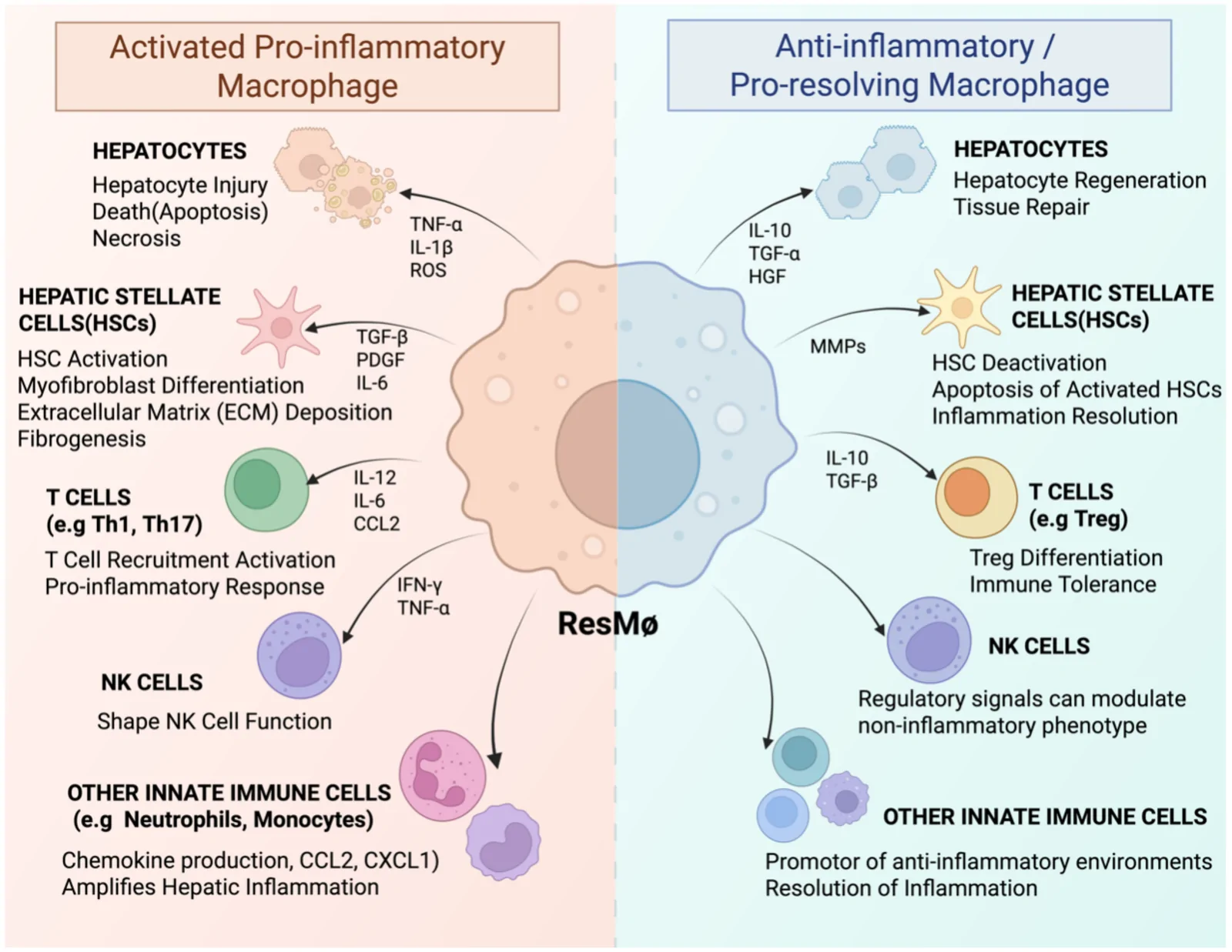
Intrahepatic intercellular communication network centered on hepatic macrophages. Bidirectional crosstalk between hepatic macrophages and other liver cells (hepatocytes, HSCs, LSECs, and adaptive immune cells). Injured hepatocytes release DAMPs to activate macrophages, while macrophages regulate hepatocyte proliferation or injury. Macrophage-derived factors promote HSC activation, whereas LSECs support ResMø quiescence. This network determines the balance between injury, fibrogenesis, and repair.

Macrophage-stellate cell crosstalk dominates fibrogenesis progression: macrophage-secreted galectin-3 and TGF-β trigger HSC trans differentiation into collagen-producing myofibroblasts, with exosomal miR-342-3p further amplifying HSC activation via HPCAL1 suppression (70, 71). LSECs maintain the quiescent state of ResMø and sinus homeostasis to limit fibrogenesis; conversely, these ResMø secrete IL-6 to promote LSEC proliferation during tissue repair. Adaptive immune cells further tune macrophage polarization: T cell-derived IFN-γ drives M1 polarization to sustain antiviral hepatic immunity (72–74).

### 4.2 Gut microbiota-macrophage axis: portal circulation-mediated systemic signaling

Gut-derived LPS and metabolites reach the liver via portal circulation. Dysbiosis compromises intestinal barrier integrity, permitting bacterial products to drive inflammatory macrophage phenotypes (75). In MASLD, LPS activates ResMø via TLR4, triggering TNF-α and IL-1β (76). Trimethylamine N-oxide (TMAO) promotes hepatic lipogenesis (77). In ALD, Gram-negative bacteria expansion leads to endotoxemia and persistent macrophage activation (78).

Reciprocally, hepatic macrophages exerts feedback regulation: IL-10 and TGF-β maintain gut barrier integrity. CX3CR1^+^ macrophages are critical for barrier function (79). This bidirectional gut-liver inflammatory loop forms a self-amplifying pathogenic cycle, which can be interrupted by probiotic, prebiotic and flavonoid interventions such as nobiletin to alleviate ALD/MASH gut-derived inflammation (80–83).

### 4.3 Autophagy, LAP and exosomes: macrophage-derived functional effectors

Autophagy and LC3-associated phagocytosis (LAP) serve as core homeostatic machineries within hepatic Mø, whereas exosomes function as secreted, cell-extrinsic mediators that transmit signals to recipient cells. These three entities must be distinguished not only by their mode of action but also by their context-dependent protective versus pathogenic roles across CLD subtypes (**Figure 5**). Autophagy exerts protective effects in MASLD via STING-YAP-dependent lipophagy that mitigates PLIN2-associated lipotoxicity, and in ALD through clearance of damaged mitochondria to constrain ROS-NLRP3 inflammasome activation. Conversely, autophagic deficiency promotes M1 polarization and perpetuates fibrogenesis (84, 85). LAP suppresses inflammation and fibrogenesis in liver fibrosis and cirrhosis through FcγRIIA/SHP-1 inhibitory signaling (86), yet assumes pathogenic functions in HCC by activating PI3Kγ/AKT in TAMs, thereby conferring resistance to PD-1 blockade (87).

**Figure 5:**
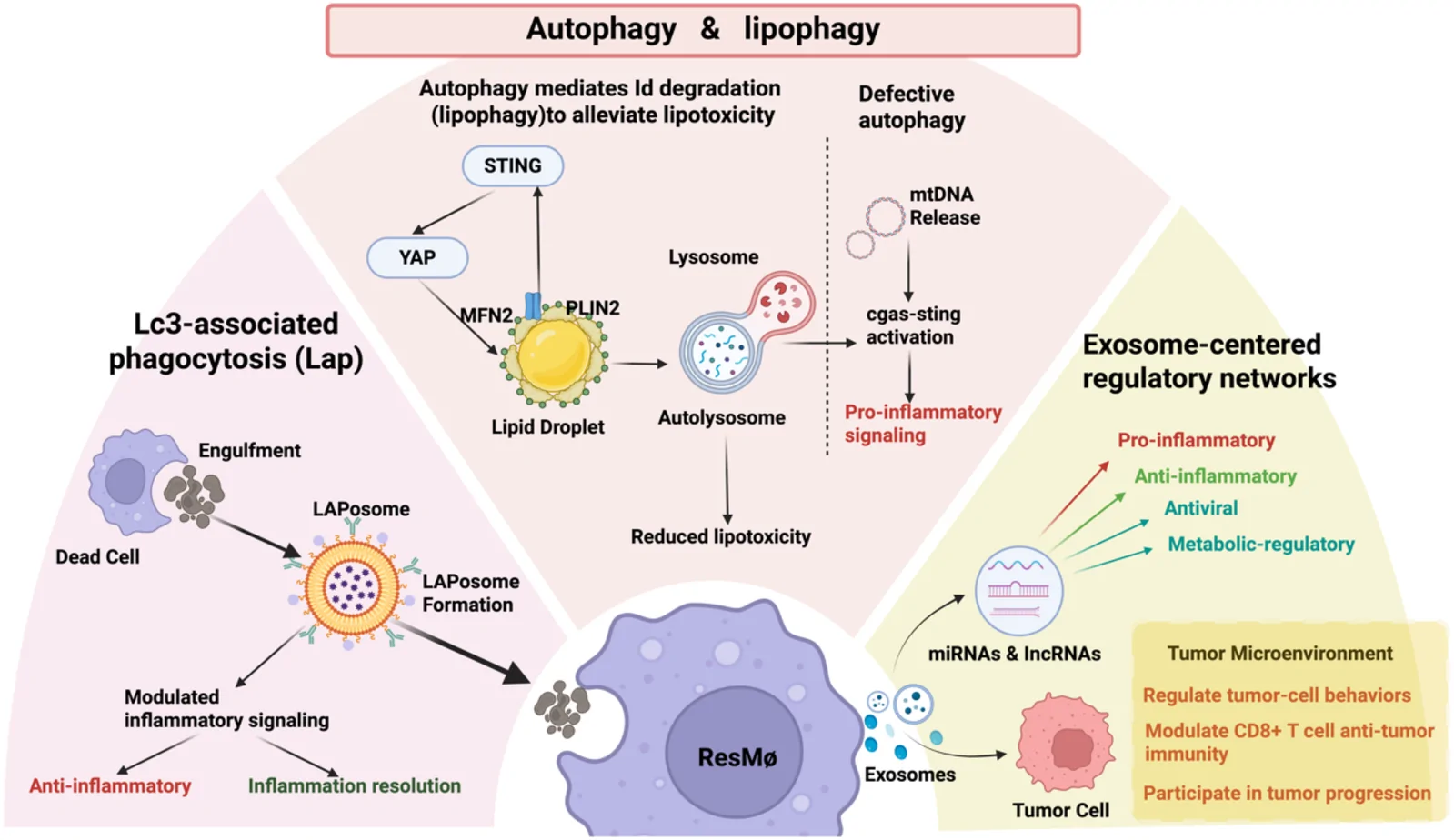
Macrophage-derived functional effectors: autophagy, LC3-associated phagocytosis, and exosomes. Three distinct effector mechanisms with context-dependent protective versus pathogenic roles. Autophagy (cell-intrinsic) protects in MASLD and ALD but promotes fibrosis when deficient. LAP (cell-intrinsic) suppresses fibrosis but promotes immunosuppression in HCC. Exosomes (secreted) predominantly drive pathology in advanced disease via lncRNA, dsRNA, and miRNA transfer. The reciprocal loop between autophagy and exosomal biogenesis is also indicated.

Exosomes are predominantly pathogenic in advanced CLD: M1-derived exosomal long non-coding RNA (lncRNA) H19 exacerbates autoimmune hepatitis via HIF-1α-p53 signaling (88); exosomal mitochondrial double-stranded RNA (dsRNA) activates TLR3 to drive IL-17A-mediated inflammation in ALD/MASLD (89); and TAM-derived exosomal miR-21-5p and miR-6876-5p promote M2 polarization, EMT, and cancer stemness via PTEN/Akt signaling in HCC (90–92), with autophagic flux and exosomal miRNAs forming a reciprocal regulatory loop that guides distinct therapeutic strategies.

## 5 Neuro-immune crosstalk along the liver-brain axis

Chronic liver injury triggers bidirectional neuro-immune communication linking hepatic macrophages activation to central neurocognitive dysfunction (**Figure 6**). The hepatic autonomic nerve network tightly regulates ResMø activity: sympathetic nerve-released norepinephrine binds macrophage β2-adrenergic receptors to boost TNF-α and IL-6 secretion, whereas parasympathetic vagal signaling inhibits NF-κB via α7 nicotinic acetylcholine receptors to restrict inflammation (93, 94). GABA neurotransmission additionally suppresses ResMø-mediated inflammatory cascades.

**Figure 6:**
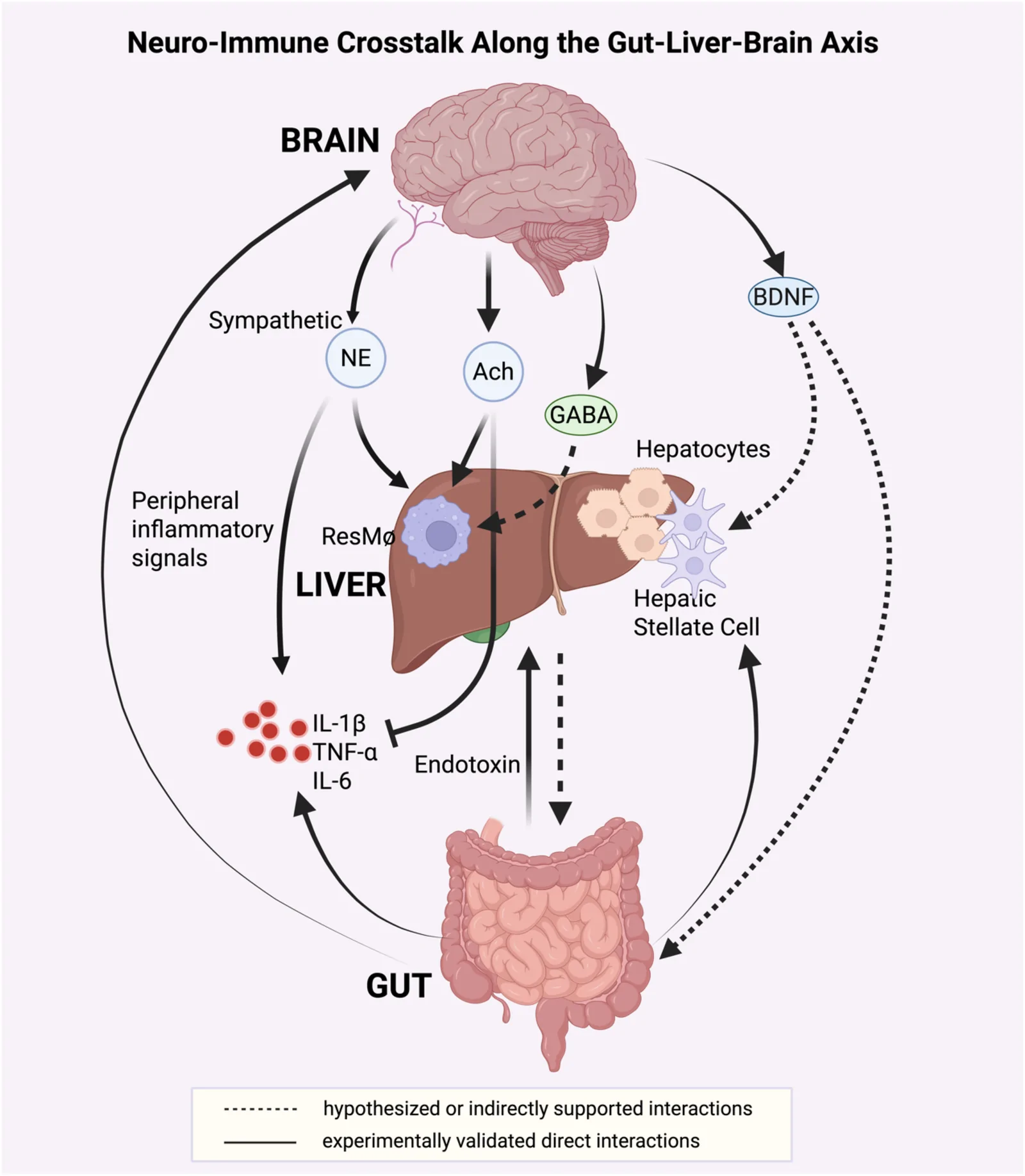
Neuro-immune crosstalk along the gut-liver-brain axis in chronic liver disease. Bidirectional neuro-immune communication integrating hepatic macrophages activation with systemic complications. Sympathetic signaling promotes while parasympathetic vagal signaling restricts inflammation. Gut microbial metabolites activate hepatic macrophages, which in turn regulate gut barrier integrity. Hepatic cytokines trigger neuroinflammation. LPAMs directly contact sympathetic nerves, and BDNF mediates protective signals. Solid arrows indicate established mechanisms; dashed arrows represent connections requiring further validation.

Hepatic inflammatory cytokines cross the blood-brain barrier to activate microglia and trigger neuroinflammation, manifesting as anxiety, cognitive decline and movement disorders in advanced cirrhosis and HCC. Gut microbial metabolites signal to the central nervous system via vagal afferent fibers, while periportal CX3CR1^+^CD63^+^ LPAMs form direct structural contacts with sympathetic nerve terminals; LPAM depletion in MASH exacerbates hepatic inflammation and concurrent neurodegeneration (16, 95, 96). Brain-derived neurotrophic factor (BDNF) mediates protective hepatic regenerative signals and limits fibrogenesis; vagus nerve stimulation elevates systemic BDNF levels to ameliorate MASLD insulin resistance and cognitive impairment (96, 97). Collectively, the gut-liver-brain neuroimmune axis positions hepatic macrophages as the central integrator of peripheral metabolic inflammation and central nervous system complications (summarized in **Table 2**).

**Table 2:** Evidence strength for neuro-immune interactions along the gut-liver-brain axis in chronic liver disease.

Neuro-immune mechanism	Proposed biological link	Study authors (PMID)	Organ/tissue studied	Relevance to CLD
Sympathetic norepinephrine–adrenergic signaling	Sympathetic norepinephrine regulates macrophage inflammatory activity through adrenergic receptors and modulates cytokine production	Mori et al. (98)	Liver; Kupffer cells	Direct evidence that norepinephrine regulates hepatic macrophage inflammatory responses
Vagal cholinergic–α7nAChR signaling	Vagal activity suppresses Kupffer cell activation through α7nAChR-dependent inhibition of inflammatory signaling	Nishio et al. (99)	Liver; Kupffer cells	Direct evidence in a NASH model supporting hepatic vagal regulation of macrophage inflammation
Vagus nerve–α7nAChR anti-inflammatory pathway	Vagus nerve stimulation suppresses macrophage cytokine production through α7nAChR-dependent signaling	Borovikova et al. (100)	Spleen; macrophages	Strong mechanistic evidence for the cholinergic anti-inflammatory pathway, but not specific to CLD
GABAergic regulation of macrophage inflammation	GABAergic signaling can modulate macrophage inflammatory responses	Cui et al. (101)	Liver; Kupffer cells	GABA ameliorates HFD-induced hepatic insulin resistance by suppressing KCs activation and its production and secretion of inflammatory cytokines.
Gut-derived microbial signals–vagal afferent pathway	Microbial-derived signals can influence CNS activity through vagal afferent pathways	Kang et al. (96)	Gut–liver-brain	Evidence largely derives from gut–liver-brain studies; direct validation in CLD remains limited
CX3CR1^+^CD63^+^ LPAM–sympathetic nerve interaction	Periportal liver portal area macrophages establish physical contacts with sympathetic nerve terminals	Xu et al. (16)	Liver; periportal LPAMs and sympathetic nerves	Direct anatomical evidence in NASH supporting a liver macrophage–sympathetic nerve interface
LPAM depletion–hepatic inflammation/nerve degeneration	Loss of LPAMs aggravates hepatic inflammation and sympathetic nerve degeneration	Xu et al. (16)	Liver; periportal LPAMs and sympathetic nerves	Direct experimental evidence linking a specialized hepatic macrophage population with both hepatic inflammation and neural integrity
BDNF–hepatocyte/hepatic steatosis	BDNF/neurotrophin signaling may regulate hepatocyte function and liver regeneration	Grzelak et al. (102)	Liver; hepatocyte	Evidence supports a liver–neurotrophin interaction, but the claim that brain-derived BDNF directly mediates hepatocyte regulation effects remains insufficiently established
Vagus nerve stimulation–BDNF–cognition	Vagus nerve stimulation can alter BDNF expression and improve cognitive function	Chunchai et al. (103)	Brain; hippocampus and cortex	Supports VNS–BDNF–cognition association, but this study was conducted in obese insulin-resistant rats rather than a CLD model

## 6 Core value, unresolved challenges and translational therapeutic perspectives

### 6.1 Integrated paradigm and conceptual innovation of this review

This work establishes a unified three-dimensional regulatory framework to systematize hepatic macrophage biology in CLD, advancing beyond the oversimplified M1/M2 binary model. Mechanistically, we consolidate metabolic reprogramming as the intrinsic master switch governing macrophage polarization, with intrahepatic intercellular communication and systemic gut-liver-brain neuroimmune signaling jointly shaping disease trajectories. Conceptually, we redefine hepatic macrophages as spatially restricted, context-sensitive functional units whose pathogenic or reparative output is jointly determined by metabolic state, anatomical localization and multi-cell microenvironmental interactions. Translationally, this review shifts therapeutic logic from non-selective pan-macrophage depletion toward precision macrophage ecosystem remodeling, highlighting biomarker stratification, targeted stroma-macrophage crosstalk intervention and emerging cell engineering platforms as next-generation CLD therapeutic avenues, consistent with TCM damp-heat clearing therapeutic strategies targeting macrophage metabolic polarization for MAFLD management.

### 6.2 Outstanding scientific barriers

Current single-cell and spatial transcriptomics datasets only capture static cellular snapshots, while macrophage migration and trans differentiation trajectories remain dynamically regulated by uncharacterized positional cues. Critical unanswered questions include the molecular drivers governing Trem2^+^ LAM migration toward fibrotic septa and conversion to SPP1^+^ SAMs, as well as how lobular zonation, stromal proximity and vascular architecture remodel macrophage effector functions. Emerging tools including multiplexed error-robust fluorescence *in situ* hybridization spatial imaging, *in vivo* spatial CRISPR screening and intravital live imaging will be required to resolve causal spatiotemporal regulatory relationships and build predictive hepatic immune ecosystem models.

Clinical translational bottlenecks persist despite promising preclinical macrophage-targeted data. The phase I MATCH01 autologous macrophage therapy trial for cirrhosis demonstrated safety yet failed primary clinical endpoints, underscoring the necessity of disease state-stratified therapeutic regimens: (1) homeostatic ResMø replenishment strategies for advanced fibrosis/cirrhosis; (2) small molecule/LNP nucleic acid macrophage reprogramming for inflammatory MASH; (3) combined TAM depletion plus immune checkpoint blockade for TAM-dominant HCC. Biomarkers including sCD163, osteopontin and circulating exosomal signatures enable patient subgroup enrichment, while combinatorial regimens with metabolic modulators (Resmetirom, FGF21 analogues) produce synergistic anti-inflammatory and anti-fibrotic effects. Complementary TCM herbal formulas targeting damp-heat syndrome provide low-toxicity alternative macrophage modulators for MASLD treatment, aligned with this review’s funded research theme.

Multi-organ neuroimmune regulation remains underexplored for therapeutic exploitation. Vagus nerve stimulation, neurotransmitter receptor modulators and blood-brain barrier permeable small molecules represent undervalued intervention strategies to disrupt pathological gut-liver-brain inflammatory loops, with hepatic macrophages serving as the central nodal therapeutic target.

### 6.3 Emerging engineered macrophage therapeutics

Beyond conventional small-molecule pharmacology, engineered macrophage therapeutics represent an emerging strategy for precision intervention in CLDs. Importantly, recent evidence has begun to move these approaches from oncology-derived concepts toward liver-specific preclinical validation. Dai et al. (104) reported that uPAR-targeted chimeric antigen receptor macrophages (uPAR-CAR-Ms) could recognize and eliminate activated hepatic stellate cells, attenuate fibrosis, and remodel the hepatic immune microenvironment in multiple mouse models of liver fibrosis and cirrhosis, providing direct proof-of-concept evidence for CAR-M therapy in liver disease. More recently, TNC-targeted CAR-Ms were shown to migrate to fibrotic livers, target TNC-expressing pathological cells, and alleviate CCl_4_-induced liver fibrosis (105). In parallel, LNP-mediated *in situ* macrophage engineering has recently demonstrated direct efficacy in liver fibrosis models. LNP delivery of CSF1 mRNA promoted the generation of fibrolytic macrophages within the liver and attenuated fibrosis in multiple mouse models, whereas CD163-targeted LNPs carrying FAP-specific CAR mRNA enabled *in situ* generation of FAP-CAR macrophages and promoted clearance of FAP-expressing pathological cells (106). These studies indicate that CAR-M and LNP-mRNA platforms have progressed from conceptual feasibility toward liver-specific preclinical validation.

Nevertheless, substantial translational barriers remain. LNP-based approaches may exhibit heterogeneous biodistribution and uptake by Kupffer cells, hepatocytes, endothelial cells, and other non-target populations, potentially reducing macrophage-selective delivery; indeed, LNP formulations themselves can activate innate immune signaling, highlighting the need to distinguish therapeutic mRNA effects from carrier-associated immunomodulation (107). Adoptively transferred CAR-Ms also face challenges related to trafficking, persistence, manufacturing, and phenotypic stability, while targets such as uPAR, TNC, and FAP may be induced during tissue injury or repair, raising potential on-target/off-target safety concerns. Microenvironment-responsive and logic-gated nanocarriers may theoretically improve spatial and temporal selectivity; however, current evidence remains predominantly derived from oncology models (108). To provide a comparative overview of these emerging engineering strategies, we summarize representative preclinical studies, their experimental contexts, target/payload design, and key translational barriers in **Table 3**.

**Table 3:** Translational evidence and challenges of engineered macrophage therapeutics for chronic liver diseases.

Engineering strategy	Representative study	Experimental context	Target/payload	Organ/model	Translational challenges
uPAR-CAR-M	Dai et al. (104)	Liver fibrosis and cirrhosis	uPAR-targeted CAR	Liver; multiple murine fibrosis models	Cell trafficking, persistence, manufacturing, target specificity
TNC-CAR-M	Chen et al. (105)	CCl_4_-induced liver fibrosis	TNC-targeted CAR	Liver; activated HSCs	Potential target expression during tissue injury/repair; long-term safety
LNP-CSF1-mRNA	Cao et al. (106)	Liver fibrosis	CSF1 mRNA	Liver; multiple murine fibrosis models	Biodistribution, transient expression, macrophage recruitment versus unwanted inflammation
CD163-targeted LNP-FAPCAR-mRNA	Huang et al. (109)	Liver fibrosis	FAP-specific CAR mRNA	Liver macrophages and fibrotic liver	Targeting specificity, LNP-associated innate immune activation, durability
AND-gated biomimetic nanoparticles	Zhang et al. (108)	Cancer immunotherapy	Dual phagocytosis-associated signals	Tumor microenvironment	No direct CLD validation; disease-specific signal selection required
Hypoxia-responsive logic-gated nanocarriers	Pan et al. (110)	Cancer models	Microenvironment-responsive payloads	Tumor tissue	Hypoxia are not fibrosis-specific; off-target activation and tissue-repair interference

Future studies should prioritize macrophage-subset-specific delivery, disease-stage-dependent targeting, quantitative biodistribution, assessment of off-target uptake and inflammatory activation, and long-term safety in clinically relevant CLD models.

## 7 Conclusion

Hepatic macrophages orchestrate CLD progression through integrated intrinsic metabolic rewiring, local intrahepatic intercellular crosstalk and systemic gut-liver-brain neuroimmune communication. To fully unlock macrophage-targeted therapeutic potential, researchers must abandon the outdated view of macrophages as isolated effector cells and instead recognize them as dynamically plastic, spatially organized signal hubs embedded within a multi-organ neuro-immuno-metabolic network. Future investigation prioritizing spatial macrophage dynamics, disease-state stratified intervention, multi-axis organ crosstalk and synthetic biological macrophage engineering will facilitate precise, individualized remodeling of hepatic immune homeostasis. This paradigm shift holds profound promise to reverse the progressive natural history of MASLD, ALD, viral hepatitis, fibrosis, cirrhosis and HCC, delivering novel actionable therapeutic strategies for global CLD patients.
